# Sepsis-Induced Cardiomyopathy: Mechanisms and Treatments

**DOI:** 10.3389/fimmu.2017.01021

**Published:** 2017-08-24

**Authors:** Yan-Cun Liu, Mu-Ming Yu, Song-Tao Shou, Yan-Fen Chai

**Affiliations:** ^1^Department of Emergency Medicine, Tianjin Medical University General Hospital, Tianjin, China

**Keywords:** sepsis-induced cardiomyopathy, mechanisms, treatments, inflammatory mediators, Chinese traditional medicine

## Abstract

Sepsis is a lethal syndrome with a high incidence and a weighty economy burden. The pathophysiology of sepsis includes inflammation, immune dysfunction, and dysfunction of coagulation, while sepsis-induced cardiomyopathy (*SIC*), defined as a global but reversible dysfunction of both sides of the heart induced by sepsis, plays a significant role in all of the aspects above in the pathogenesis of sepsis. The complex pathogenesis of *SIC* involves a combination of dysregulation of inflammatory mediators, mitochondrial dysfunction, oxidative stress, disorder of calcium regulation, autonomic nervous system dysregulation, and endothelial dysfunction. The treatments for *SIC* include the signal pathway intervention, Chinese traditional medicine, and other specific therapy. Here, we reviewed the latest literatures on the mechanisms and treatments of *SIC* and hope to provide further insights to researchers and create a new road for the therapy of sepsis.

## Introduction

Sepsis is a lethal syndrome induced by infection, which has a reported annual death of 200,000 in the United States (1). The pathophysiology of sepsis includes inflammation, immune dysfunction, and coagulation disorders. Though studies have confirmed the immunosuppression in the late stage as the leading cause of mortality of septic patients; however, the septic shock in the early onset of sepsis, which induced by cytokine storm and cardiac dysfunction, is also an important cause of death for septic patients, especially for the young patients with toxic shock syndrome or meningococcemia (2).

Heart, as the pump organ, plays a key role in the pathology of septic shock. With the development of tissue Doppler imaging, perfusion echocardiography myocardial, and hemodynamics monitoring (3), the definition of sepsis-induced cardiomyopathy (*SIC*) has been summarized as a global (systolic and diastolic) but reversible dysfunction of both the left and right sides of the heart, which is induced by myocardial depressants released from pathogen and host, and global ischemia after peripheral vasodilation, arterial and capillary shunting in septic distributive shock (4). A retrospective cohort study reported that *SIC* developed in 13.8% of patients with sepsis and septic shock (5), which could be used as an outcome predictor in the septic patients (6). The mechanisms of *SIC* includes the preload deficiency during distributive shock, heart–lung interactions during mechanical ventilation, and ventricular–arterial coupling in the presence of vasopressors (7); however, here, we focus on the mechanisms of cardiomyocyte dysfunction and treatments of *SIC* and hope to provide further insights for the management of *SIC*.

## The Mechanisms of SIC

### Pathogen-Associated Molecular Patterns (PAMPs)

Pathogen-associated molecular patterns released by infecting organisms not only bind immune receptors on inflammatory cells but also bind receptors on cells in the heart (8). Endotoxin is released by the lysis of Gram-negative bacteria. A research from healthy volunteers showed that there was a reduction in left ventricular ejection fraction (LVEF) and LV performance after the injection of endotoxin (9). The endotoxin-induced myocardial dysfunction probably depends on the toll-like receptor (TLR) 4-induced cytokines release as a delay in onset of myocardial depression after endotoxin administration (10). Circulating Pneumolysin, another common PAMP produced by *Streptococcus pneumoniae*, induced cardiomyocyte injury through triggering profound calcium influx during pneumococcal infection (11).

### Toll-Like Receptors

Toll-like receptors are transmembrane glycoproteins, which recognize many PAMPs with extracellular domains and aggravate the exaggerated inflammatory response to bacterial infection through activating nuclear factor (NF)-κB (12). TLR4 is the most studied member in the *SIC* study among the TLRs family. A research from TLR4-deficient mice confirmed the essential role of TLR4 in mediating neutrophil migratory phagocytic functions, attenuating inflammation, reducing reactive oxygen species generation, and enhancing bacterial clearance (13). Other TLR-related genes (TLR2, 3, and 9) were demonstrated to be involved in sepsis-induced cardiac dysfunction from recent studies. TLR2 increased the myocardium and serum cardiodepressant cytokines level and weakened the neutrophil migratory function, which sharpened the *SIC* (14). TLR3 played a deleterious role in mediating cardiac dysfunction in sepsis by increasing cecal ligation and puncture (CLP)-induced cardiomyocytes apoptosis and Fas and Fas ligand expression in the myocardium (15). CpG oligodeoxynucleotide, the TLR9 ligand, through activating both phosphoinositide 3 kinase/Akt and extracellular signal-related kinase signaling, attenuated cardiac dysfunction in polymicrobial sepsis (16). However, a recent research demonstrated that eritoran, an anti-TLR4 to terminate MD2/TLR4-mediated signaling, did not significantly improve outcome for patients with severe sepsis and septic shock (17). Additional studies are needed to explain the detailed mechanisms of *SIC* regulated by TLRs.

### Cytokines

The main inflammatory mediators that might contribute to *SIC* are tumor necrosis factor (TNF)-α, interleukin (IL)-1, and IL-6. In 1992, Eichenholz et al. demonstrated that TNF-α in plasma had a dose-dependent relationship with the depression of LVEF from a canine model of septic shock (18). At the same year, Vincent et al. showed that the administration of murine monoclonal anti-TNF antibodies could transiently improve ventricular function in patients with septic shock (19). IL-1, cooperated with TNF-α, decreased the myocardial contractility, and played an active role in septic myocardial dysfunction. In a recent study from children with meningococcal septic shock (20), Pathan et al. confirmed the negative inotropic effects created by IL-6 *via* a p38 mitogen-activated protein kinase pathway and suggested a novel therapy to reverse myocardial dysfunction. However, as the half-lives of TNF-α, IL-1, and IL-6 are less than 6 h, it seemed that these cytokines did not induce *SIC* independently, and the detailed mechanisms still need further investigations.

### Damage-Associated Molecular Patterns (DAMPs)

#### Extracellular Histones

Circulating extracellular histones in plasma have been detected in septic patients, and it interacts with TLR2 and TLR4 on a variety of different cell types and contributes to endothelial dysfunction, organ failure, and death in experimental sepsis (21, 22). A recent study showed that high levels of histones in plasma were significantly associated with cardiac injury and dysfunction in septic patients and high level of histones predicted a worse outcome (23), and another study from septic mice demonstrated that neutralizing histones antibodies or drugs, which block histones interactions with cardiomyocytes might represent an effective strategy to prevent or ameliorate *SIC* (24). However, it is unclear that whether the histones in plasma are the cause or the result of *SIC* as histones occur inside the nucleus and can be released into circulation because of cytokines storm and cellular death during sepsis. Further researches are needed to confirm the exactly roles played by histones in the pathogenesis of *SIC*.

#### Heat Shock Proteins (HSPs)

Heat shock proteins, a group of highly selective proteins produced by cells in reaction to stress, appear to play a critical role in the development of thermotolerance and protection from cellular damage associated with stresses (25). Early research has shown that HSP72 can reverse the cardiac dysfunction of septic model induced by CLP (26). Some researches subsequently unveiled that HSP20-attenuated endotoxin-induced myocardial dysfunction and apoptosis *via* inhibition of NF-κB activation (27), while HSPA12B reduced the leukocytes infiltration into the myocardium and prevented *SIC* through preserving activation of PI3K/Akt signaling (28). Later, Hsu et al. confirmed that exogenous HSC70 pretreatment attenuated LPS-induced myocardial dysfunction in septic rats (29). The detailed protective mechanisms of HSPs in sepsis still require further studies.

#### High-Mobility Group Protein B (HMGB)1

High-mobility group protein B1, a non-histone nuclear protein, serves as an alarmin to drive the pathogenesis of inflammatory and autoimmune disease (30). The serum level of HMGB1 in septic patients induced by severely burn is significantly increasing, and it is associated with the fatal outcome of sepsis (31). Xu et al. demonstrated that HMGB1 could also be produced by viable cardiomyocytes in septic mice, and it mediated the LPS-induced myocardial dysfunction through a TLR4/PI3Kγ signaling pathway (32). Zhang et al. showed that HMGB1 enhanced sarcoplasmic reticulum Ca^2+^ leak through TLR4-reactive oxygen species signaling pathway, and decreased systolic Ca^2+^ transient and cardiomyocytes contractility, both contribute to the mechanisms underlying the HMGB1-induced *SIC* (33).

### Nitric Oxide (NO)

Nitric oxide, a small and highly reactive molecule with a half-life of a few seconds, is produced from all types of cardiac cells and has a multitude of cardiovascular effects in cardiovascular homeostasis. NO is produced from nitric oxide synthese (NOS), which exists in two forms: constitutive (NOS1, NOS3) and inducible (NOS2). NOS1 and NOS3 relevantly contribute to bioactive NO pool (34) and culprit in early septic myocardial depression, whereas NOS2 may mediate myocardial depression that occurs in late sepsis (35). The mechanisms of NO-induced *SIC* include vasodilatation with resulting changes in preload, afterload, and cardiac perfusion, downregulating β-adrenergic receptors (36), depression of mitochondrial respiration, and further release of pro-inflammatory cytokines (37). Another adverse effect of NO on myocardial depression is the peroxynitrite, produced by NO metabolism, which interacts with lipids, DNA, and proteins (38) and affects the function of mitochondrial permeability transition pores, with subsequent mitochondrial dysfunction (39). Removal of peroxynitrate improved the myocardial performance in the study of cytokine-induced myocardial depression (40).

### Mitochondrial Dysfunction and Oxidative Stress

Mitochondrial dysfunction plays a significant role in the pathogenesis of sepsis and the degree of mitochondrial dysfunction is correlated with outcomes. Cardiomyocytes demonstrate mitochondrial ultrastructural damage in both septic animals and patients (41). Studies have shown that non-competitive inhibition of cytochrome C oxidase developed during sepsis, which interrupted the inhibition of oxidative phosphorylation and decreased production of ATP, leading to sepsis-associated myocardial depression (42). Low T3 syndrome, a very common phenomenon in septic patients, could also induce the mitochondrial dysfunction and sharpen the myocardial depression (43). When mitochondrial dysfunction persisted, ROS were generated in cardiomyocytes from septic heart, while oxidative stress induced by ROS mediated mitochondrial damage, which accelerated the mitochondrial dysfunction (44). The administration of superoxide scavenger compounds (44) and the inhibition of mitochondrial dysfunction (45) have been shown to prevent mitochondrial abnormalities and improve cardiac function and reduce mortality.

### Disorders of Calcium Regulation

In the normal state, extracellular calcium enters the cardiomyocytes *via* L-type channels and releases intracellular calcium from sarcoplasmic reticulum, which binds to troponin and activates the contraction proceeds with ATP hydrolysis (46). In the complex situation of SIC, the density of calcium L-type channels decreased, calcium sequestration is in disorder as the phosphorylation of phospholamban is in turbulence, and myofilament calcium sensitivity and responsiveness of the ryanodine receptor to calcium reduced (47). Furthermore, DAMPs, like HMGB-1, enhances SR calcium leak through the TLR4–ROS signaling pathway, then calcium transients and cardiomyocytes contractility decreased. Hence, inhibiting TLR4 or using antioxidant prevents the enhancement of the SR calcium leak, resulting in alleviating myocardial dysfunction (48).

### Autonomic Nervous System Dysregulation

**Figure 1:**
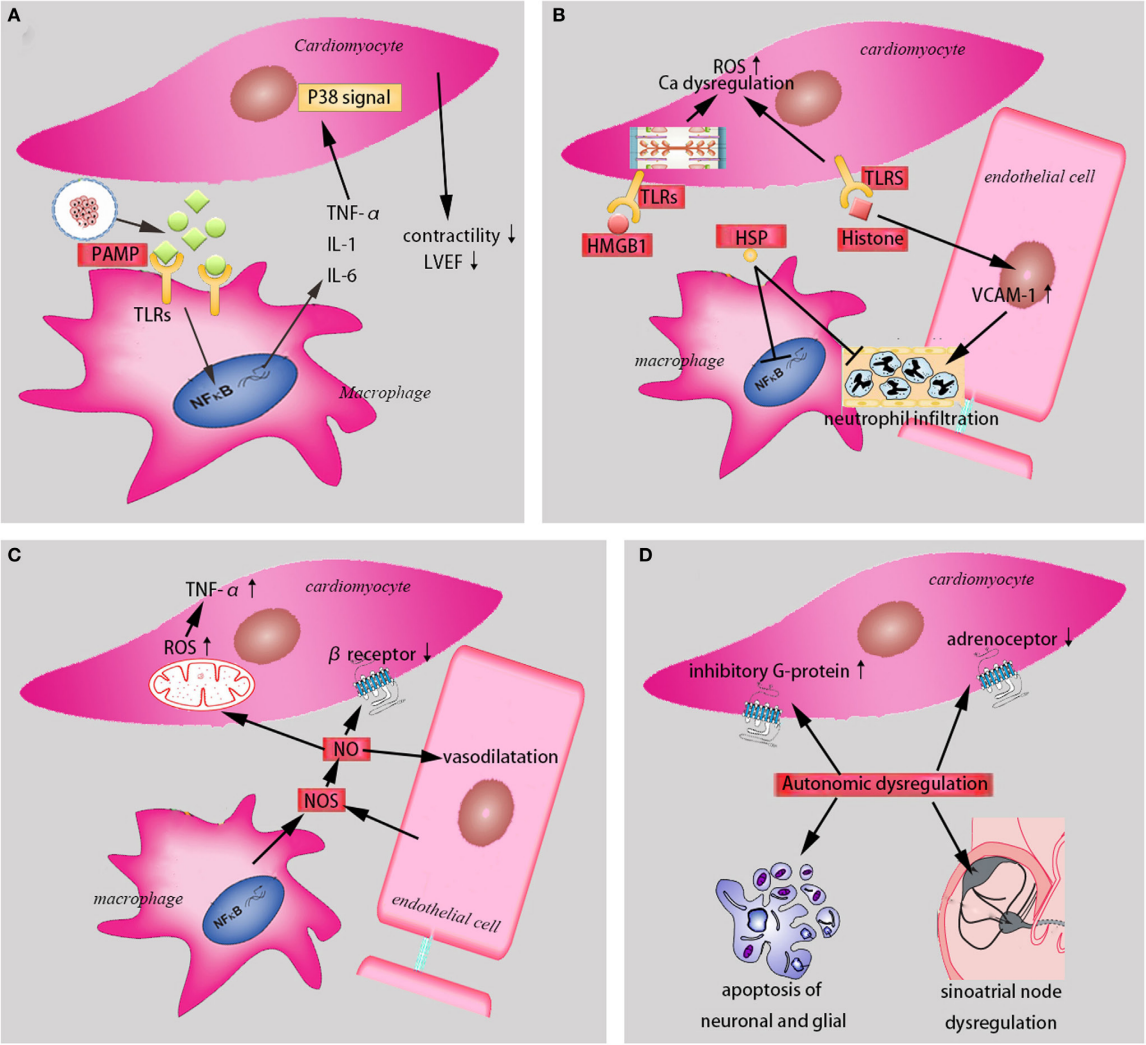
The mechanisms in the sepsis-induced cardiomyopathy. **(A)** PAMPs and cytokines (TNF-α, IL-1, and IL-6) contribute to sepsis-induced cardiomyopathy (*SIC)*. **(B)** Damage-associated molecular patterns (HMGB1, HSP, and histone) induce *SIC* through different mechanisms. **(C)** NO and NOS are involved in *SIC*. **(D)** Autonomic dysregulation play a significant role in *SIC*. PAMP, pathogen-associated molecular pattern. TNF-α, tumor necrosis factor-α. IL-1, interleukin-1. IL-6, interleukin-6. TLRs, toll-like receptors. NF-κB, nuclear factor-κB. LVEF, left ventricular ejection fraction. HMGB1, high mobility group protein B1. HSP, heat shock protein. ROS, reactive oxygen species. VCAM, vascular cell adhesion molecule. NO, nitric oxide. NOS, nitric oxide syntheses.

Autonomic dysregulation, which includes resistance to catecholamines and the loss of heart rate variability in septic state plays a significant role in the sepsis-induced cardiac depression. Some studies have demonstrated that septic animal performance a state of catecholamines resistance, which was mediated by the decreased density of myocardial adrenoceptors, the increased expression of inhibitory G-protein, and the disrupted signal transduction (49), despite elevated circulating levels of catecholamines. Hoyer et al. (50) confirmed that the loss of heart rate variability indicated a high probability of progression to multiple organs failure with poor outcomes. When sepsis occurred, apoptosis of neuronal and glial within cardiac autonomic centers increased (51), sepsis-induced uncoupling of the sinoatrial node from cholinergic neural control (52), and the direct current blockade of the sinoatrial node by the elevated cytokines of plasma (53), all contributed to the loss of heart rate variability (Figure 1).

### Endothelial Dysfunction

Sepsis induces the endothelial abnormalities, which is another important aspect that leading to *SIC*. Endothelial cells secrete more adhesion molecules, which assist injurious neutrophilic infiltration to cardiomyocytes and increase the leukocyte–endothelium interaction in response to inflammatory cytokines in sepsis (54). Vascular cell adhesion molecule 1 (VCAM-1), as the most common example, has been demonstrated in the coronary endothelium and cardiomyocytes of murine models of septic shock. Blockade of VCAM-1 abrogates neutrophil accumulation and prevents the cardiac dysfunction in sepsis (55). Calpain is over activated in cardiac tissue in the setting of sepsis, and the inhibition of Calpain decreases the leukocyte–endothelium interaction and improves sepsis-induced cardiac dysfunction (56). Both of them suggest that VCAM-1 and Calpain may serve as therapeutic targets for *SIC*. Physical disruption of endothelial cells is another mechanism for the *SIC*. The injured endothelial cells secrete intercellular adhesion molecule, E-selectin, and thrombomodulin, which lead to the failure of vascular relaxation and are prognostically related to cardiac systolic and diastolic dysfunction (57).

## The Treatments of SIC

### Hemodynamic Stabilization

When septic shock occurs, PAMPs, DAMPs, oxidative stress, mitochondrial dysfunction, disorders of calcium regulation, autonomic nervous system dysregulation, and endothelial dysfunction all contribute to the pathogenesis of *SIC*. On the other hand, the consequence of *SIC* is an insufficient supply of oxygen to meet tissue demand, which causes global tissue hypoxia and accelerates the process of septic shock (58). Thus, focusing on hemodynamic stabilization is the foremost step in patients with *SIC* apart from the proper management of infection.

Rapid and effective fluid therapy guided by monitoring fluid response parameters to remedy hypovolemia is recommended as the cornerstone of sepsis treatments (58). Crystalloids is recommended as the initial fluid in the resuscitation of sepsis; however, a research from septic animal demonstrated that albumin and hypertonic saline were more beneficial on cardiac function compared with normal saline (59).

Inotropic drugs are suggested for the septic patients with low cardiac output after optimization of fluid therapy. Norepinephrine is the first choice from the current guidance, while dobutamine and dopamine are only used in highly selected patients. The use of dopamine was associated with a greater number of adverse events (60). Levosimendan, a calcium-sensitizing drug, sensitizes troponin C to calcium and enhances the effects of calcium on myofilaments during contraction. Different with dobutamine and dopamine, levosimendan does not stimulate β-adrenergic receptor and cause adverse effects. Studies have shown that levosimendan could preserve septic heart function through cooling down the oxidative burst of circulating cells and inhibiting the release of reactive oxygen species (61). However, a recently randomized clinical trial showed that the addition of levosimendan in adult with sepsis was not associated with lower mortality, and it decreased the probability of successful weaning from mechanical ventilation and increased the risk of supraventricular tachyarrhythmia (62).

### Signaling Pathways Intervention

Multiple signaling pathways are involved in the pathogenesis of *SIC*, and therapies of targeting signaling pathways have been researched always. Annexin A5, a 35-kDa phospholipid binding protein, decreases cytokine expression and improves cardiac function during endotoxemia treatment through inhibiting LPS binding to TLR4 and leading to reductions in mitogen-activated protein kinase and Akt signaling (63). Peroxisome proliferator-activated receptor (PPAR), a nuclear receptor, regulates cardiac fatty acid oxidation. Drosatos et al. demonstrated that activation of PPARγ prevented cardiac dysfunction and mortality in spite of development of cardiac inflammation and downregulation of PPARα in LPS-treated mice (64). Neuregulin-1 (NRG-1), a member of the family of epidermal growth factors, improved cardiac function and protected cardiomyocytes of rats from sepsis *via* the activation of NRG-1/ErbB signaling axis (65). However, there are some difficulties present in this treatment strategy. First, almost all attempts from signaling pathways intervention are researched from animal models. Second, a single pathway possesses a lot of biological activities. Third, the related pathways influence each other through a complex network of regulatory interactions. All these difficulties remind us the therapy to *SIC* from signaling pathways intervention still have a long way to go.

### Traditional Chinese Medicine (TCM)

Traditional Chinese medicine has been used for treatment of sepsis in China for many years, and some of them have been confirmed to have beneficial effects for the *SIC*. Paeoniflorin, one of the major bioactive components of paeony root, attenuates cardiac dysfunction in septic mice *via* the inhibition of NF-κB (66). The salutary effects of resveratrol rescue animals from *SIC* through reversing sepsis-dependent downregulation of PPARγ co-activator 1α, and preserving mitochondrial integrity (67). Xuebijing injection (XBJ) is a complex traditional prescription, which is extracted from several herbs with immune modulating functions. XBJ has a significant efficacy in the therapy of sepsis through promoting septic macrophage polarization (68), which has the potential effect in regulating cardiac function in sepsis. With the quick development of pharmaceutical analysis, more and more TCM were analyzed and the detailed mechanisms were gradually revealed, which has a great potential for the researches of *SIC*.

### Others

β-adrenoreceptor antagonist has been rarely used in the treatment of patients with septic shock as the existence of *SIC*. It will potentially decrease the blood pressure, perform negative inotropic effects, and cause pump failure in the already depressed heart. Recently, with the mechanisms of pathological catecholamine excess in *SIC* was revealed, increasing experimental evidences have suggested that β-adrenergic regulation may improve cardiac function during septic shock (69). The use of esmolol was associated with reduction of heart rates, and no increased adverse events in septic shock patients (70). Adjunction of selective β1-blockade enhances intrinsic cardiac contractility and vascular responsiveness to catecholamine through anti-inflammatory, lowers heart rate for better ventricular filling during diastole, and performs cardiac protective effects in septic animals (71). However, more large clinical trials with different risk subsets and timing of administration are needed to confirm its effects.

Erythropoietin (EPO) is widely used for the treatment of anemia in patients, especially the anemia in patients with chronic kidney disease. The administration of EPO attenuated the impaired systolic contractility in experimental sepsis *via* activation of the β-common receptor (72). As the complex and strong interaction between EPO and βcR (73), selectively activating the tissue-protective βcR-EpoR heterocomplex represents a new therapeutic approach to *SIC*.

microRNAs (miRNAs) are a class of small 21–23 nucleotides long RNAs molecules, which have been identified as novel regulators of gene expression at the posttranscriptional level. miRNAs play strong regulatory effect in almost all cardiovascular processes, like myocardial infarction and heart failure phases (74). Recent studies have shown that miRNAs played a critical role in sepsis-induced cardiac dysfunction. Gao et al. used lentivirus-expressing miR-146a to transfect before subjecting to CLP and results showed that miR-146a attenuated *SIC* by preventing sepsis-induced NF-κB activity, attenuating inflammatory cytokine production and decreasing sepsis-induced neutrophils infiltration and macrophages into the myocardium (75). Wang et al. used LPS to induce *SIC* and provided strong evidence that miR-21-3p controlled *SIC via* regulating SH3 domain-containing protein 2 and inhibition of miR-21-3p might be a potential strategy to treat *SIC* (76). It can be seen that miRNAs-targeting therapy might open a new era for the treatment of *SIC* (Table 1).

**Table 1:** Treatments, mechanisms, and types of study of sepsis-induced cardiomyopathy.

Treatments	Mechanisms	Study type	Details of sepsis	No. of sepsis/total (%)	Reference
Levosimendan	Calcium-sensitizing, inhibiting reactive oxygen species	Critically ill patients	Clinical diagnosis of sepsis	9/25 (36)	(59)
Annexin A5	Binding to TLR4, mitogen-activated protein kinase and Akt signaling↓	Animal	Intraperitoneal endotoxin	52/103 (50)	(63)
Peroxisome proliferator-activated receptor (PPAR)γ	Activation of PPARγ	Animal	Intraperitoneal endotoxin	14/28 (50)	(64)
Neuregulin-1 (NRG-1)	NRG-1/ErbB↑	Animal	Cecal ligation and puncture (CLP)	22/27 (81)	(65)
Paeoniflorin	NF-κB↓	Animal	intraperitoneal endotoxin	24/48 (50)	(66)
Selective β1-blockade	NF-κB↓	Animal	CLP	18/24 (75)	(71)
EPO	β-common receptor activation	Animal	Intraperitoneal endotoxin, CLP	112/153 (73)	(72)
miR-146a	NF-κB↓	Animal	CLP	12/24 (50)	(75)
miR-21-3p	Regulating SH3 domain-containing protein 2	Animal	Intraperitoneal endotoxin	12/24 (50)	(76)

## Conclusion and Perspective

Sepsis-induced cardiomyopathy has been defined as a global but reversible dysfunction of heart, and it has been always the topic of intensive research for last four decades. The complex pathogenesis of *SIC* involves a combination of dysregulation of inflammatory mediators, mitochondrial dysfunction and oxidative stress, disorder of calcium regulation, autonomic nervous system dysregulation, and endothelial dysfunction. Although much progress has been made in the therapies of *SIC*, like signaling pathways intervention, TCM, β-adrenoreceptor antagonist, EPO, and microRNA, there is still no efficient treatment in patients with *SIC*.

## Ethics Statement

The study was approved by the ethics committees at the Tianjin Medical University General Hospital, Tianjin, China.

## Author Contributions

Y-CL and M-MY drafted the manuscript and performed a literature review. S-TS and Y-FC were served as chief physicians. All authors read and approved the final manuscript.

## Conflict of Interest Statement

The authors declare that the research was conducted in the absence of any commercial or financial relationships that could be construed as a potential conflict of interest.

## Abbreviations

*SIC*, sepsis-induced cardiomyopathy; TDI, tissue Doppler imaging; PAMPs, pathogen-associated molecular patterns; TLR, toll-like receptor; TNF, tumor necrosis factor; IL, interleukin; LVEF, left ventricular ejection fraction; HSPs, heat shock proteins; CLP, cecal ligation and puncture; HMGB, high-mobility group protein B; NO, nitric oxide; NOS, nitric oxide synthese; DAMPs, damage-associated molecular patterns; VCAM-1, vascular cell adhesion molecule 1; ICAM, intercellular adhesion molecule; PPAR, peroxisome proliferator-activated receptor; NRG-1, neuregulin-1; TCM, Traditional Chinese medicine; XBJ, Xuebijing injection; EPO, erythropoietin; miRNAs, microRNAs.
